# A Comparison

**Published:** 1913-07

**Authors:** 


### A Comparison.

One of the characteristics of the present-day campaign for the prevention of disease is the homely, practical way in which facts are being placed before the public. Many of our State boards, through bulletins, are doing excellent work in this direction. As a result, some popular ideas are being sadly shaken. The little housefly, for instance, has been for years the subject of household poetry and has been referred to as the harmless and innocent companion of man. The bedbug, on the contrary, has been looked on with speechless aversion. He has no social standing. Even the mention of his name has not been considered good form in our best circles, while the least suspicion of a speaking acquaintance with him has been regarded with horror. In the May number of the Bulletin of the North Carolina State Board of Health, Dr. Cyrus Thompson, in an article on "Flies and Filth," says:

"Now, as a matter of unprejudiced fact, barring the sting of the bite and the odor of the encounter, the bedbug is a much more eligible companion than the housefly, whether of bed or of board. But if bedbugs, comparatively cleanly of habit, crawled all over our plates, table and food just as the houseflies crawl, fresh from the foulest filth of every pestilential kind, who could eat or even sit at the table for a moment? I am not making a plea for the elevation of the social status of my nocturnal friend, who loves darkness rather than light; but I am declaring that his deeds are not nearly so evil and destructive as those of the housefly."

Put this statement before every American housekeeper and the doom of the typhoid fly is sealed. The bedbug has been for generations the abomination of the housewife and the object of her unrelenting warfare. Once convince American women that the fly is more loathsome and dangerous than the bedbug and the ravages of this typhoid-breeder and filth-distributor will be over.—*Exchange.*

